# Prevalence of Multidrug-Resistant Foodborne Pathogens and Indicator Bacteria from Edible Offal and Muscle Meats in Nashville, Tennessee

**DOI:** 10.3390/foods9091190

**Published:** 2020-08-28

**Authors:** Siqin Liu, Agnes Kilonzo-Nthenge, Samuel N. Nahashon, Bharat Pokharel, Abdullah Ibn Mafiz, Maureen Nzomo

**Affiliations:** 1 Department of Agriculture and Environmental Sciences, Tennessee State University, 3500 John A. Merritt Boulevard, Nashville, TN 37209, USA; sliu@tnstate.edu (S.L.); snahashon@tnstate.edu (S.N.N.); bpokhare@TNState.edu (B.P.); amafiz@tnstate.edu (A.I.M.); mnzomo@tnstate.edu (M.N.); 2 Department of Human Sciences, Tennessee State University, 3500 John A. Merritt Boulevard, Nashville, TN 37209, USA

**Keywords:** antimicrobial resistance, edible offal, muscle meat

## Abstract

This study investigated the prevalence of antimicrobial-resistant bacteria in retail edible offal and muscle meats in Nashville, Tennessee. A total of 348 retail meats (160 edible offal and 188 muscle) were analyzed for *Salmonella enterica* serovar, *Campylobacter*, *Escherichia coli*, *E. coli* O157:H7, and enterococci. Bacteria was identified using biochemical and PCR methods. *Salmonella enterica* serovar (4.4% and 4.3%), *Campylobacter* (1.9% and 1.1%), *E. coli* (79.4% and 89.4%), and enterococci (88.1% and 95.7%) was detected in offal and muscle meats, respectively. Chicken liver (9.7%) was most frequently contaminated with *Salmonella enterica* serovar, followed by ground chicken (6.9%) and chicken wings (4.2%). No *Salmonella enterica* serovar was detected in beef liver, beef tripe, and ground beef. The prevalence of *Campylobacter* was 6.9%, 2.3%, and 1.4% in beef liver, ground beef, and ground chicken, respectively. None of the meats were positive for *E. coli* O157:H7. Resistance of isolates was significantly (*p* < 0.05) highest in erythromycin (98.3%; 99.1%), followed by tetracycline (94%; 98.3%), vancomycin (88.8%; 92.2%) as compared to chloramphenicol (43.1%; 53.9%), amoxicillin/clavulanic (43.5%; 45.7%), and ciprofloxacin (45.7%; 55.7%) in offal and muscle meats, respectively. Imipenem showed the lowest resistance (0%; 0.9%). A total of 41 multidrug-resistant patterns were displayed. Edible offal could be a source of antibiotic-resistant bacteria.

## 1. Introduction

American’s food demographics continue to adjust as a result of an expanding U.S. population of immigrants [1]. Immigrants maintain their food traditions in the United States, permitting them to continue with their culture, structure, and identity. Western culture including food selection has been influenced by engaging in cultural festivities and activities of immigrants. There is also a long-standing impact of immigration on American cuisine. Several edible offal including fried chopped liver, giblet gravy, liver mush, and gandinga in the U.S. are good examples of dishes which have emerged and embraced as a result of immigration. Edible offal, as well as muscle meats, are consumed for their nutritional benefits [2]. However, undercooked meats are one of the leading origins of foodborne diseases [3]. Meats are frequently contaminated with zoonotic pathogens all through production, processing, distribution, and retail processing [4].

*Salmonella enterica* serovar, *Campylobacter*, *E. coli* O157:H7 among others are the most shared foodborne pathogens of animal origin [5]. *Escherichia coli* and enterococci are also common in meat products and reflect the microbial quality of the environment [6]. *Escherichia coli, Enterococcus faecalis,* and *Enterococcus faecium* are habitants of the environment and animal guts; all associated with gastrointestinal infections and also a measure of fecal contamination [7,8]. Edible offal spoil fast owing to their functional structures and are often tainted with pathogenic bacteria [9]. Some parts of the world prepare slightly cooked offal dishes, a practice that may cause foodborne illnesses. Campylobacteriosis was reported in New Zealand and the U.S. due to consumption of undercooked liver [10]. Often restaurants serve undercooked liver to retain nutritional benefits, which could cause illness as liver tissue may be contaminated with *Campylobacter* [11] and other foodborne pathogens. Organs including liver and tripe should be prepared at a minimum internal temperature of 160 °F (71.1 °C).

Antimicrobial resistance in food animals is a major concern due to the potential dissemination of resistant bacteria to humans via the food chain [12]. According to Mcewen and Fedorka-Cray [13], antimicrobial-resistant bacteria may leak and contaminate meat during slaughtering and could transfer to humans through food. Bosilevac et al. [14] report that meats are a cradle and vehicle for dispersion of antibiotic-resistant bacteria to humans. Since edible offal has received considerable focus due to its nutritious qualities [15], it is essential to evaluate their microbiological quality. Therefore, this study assessed the prevalence of antimicrobial-resistant bacteria from retailed offal and muscle meats in Nashville, Tennessee. Nashville is a city with an influx of immigrants and notably, some chain retail stores have offal sections to accommodate individuals embracing offal-based dishes. Again, Nashville is in the southern U.S., also accommodating use in traditional southern foods.

## 2. Materials and Methods

### 2.1. Sample Collection and Preparation

A total of 160 edible offal (chicken liver = 72; beef liver = 44; and beef tripe = 44) and 188 muscle meats (chicken wing = 72; ground chicken = 72; and ground beef = 44) were purchased from three retail stores in Nashville, over 6 months from August 2017 to January 2018. Meats per sample type were purchased in duplicates at each selected store and identified as MSA (*n* = 2), MSB (*n* = 2), and MSC (*n* = 2) to keep their confidentiality. In total, meat samples were purchased from 6 stores. The number of meat samples purchased each week was dependent on availability at the time of collection. All meats were acquired before the best-selling date, labeled with store identification letter, and date of collection. Meats were transported in iceboxes to the laboratory and processed within 2 h of collection. From each meat sample, 3 sub-samples were prepared and processed for bacterial isolation and identification. Concisely, 25 g of each sub-sample were aseptically placed in a sterile stomacher bag (Fisher scientific, Pittsburgh, PA USA) into which 225 mL sterile buffered peptone water (BPW) (Oxoid, Solon, OH, USA) was added. The mixture was homogenized (Stomacher^®^ 400 Circulator, Seward, Norfolk, UK) at 230 rpm for 2 min. Prevalence of *Salmonella enterica* serovar, *Campylobacter*, *Escherichia coli* O157:H7, generic *Escherichia coli*, and enterococci were evaluated from all homogenized samples.

#### 2.1.1. Detection of *Salmonella Enterica* serovar

For pre-enrichment, the homogenized mixture was incubated for 24 h at 37 °C. Approximately 1 and 0.1 mL of each pre-enriched sample was added to 10 mL of Tetrathionate (TT) broth (BD, Franklin Lakes, NJ, USA) and Rappaport-Vassiliadis (RV) broth (BD, Franklin Lakes, NJ, USA), respectively. The TT and RV enrichment cultures were incubated at 37 °C and 42 °C, respectively for 24 h. After enrichment, 10 μL of each sample was streaked in duplicates onto xylose lysine Tergitol 4 (XLT-4; Oxoid) and CHROMagar Salmonella (CAS) agar (Oxoid) plates. After incubation at 37 °C for 24 h, colonies that were red to yellow with black centers on XLT-4 or mauve (rose to purple) on CAS plates were identified as presumptive *Salmonella enterica* serovar. Oxidase and *Salmonella enterica* serovar agglutination tests (FT0203; Oxoid) were additionally used for further identification of *Salmonella enterica* serovar. All presumptive isolates were tested biochemically by using API 20E strips (bioMérieux, Hazelwood, MO, USA). *Escherichia coli* ATCC 25922, *Klebsiella pneumoniae* and *pneumoniae* ATCC 35657 were used as quality strains. Three colonies per plate were selected for API biochemical testing. The isolates with >90% confidence level identification were confirmed by PCR.

#### 2.1.2. Detection of *Campylobacter* spp.

For each meat sample, 1 mL of the homogenized mixture was enriched in 9 mL of sterile Bolton Broth (CM 0983; Oxoid, Solon, OH, USA) with CCDA Lysed Horse Blood (SR0048C; Oxoid, Solon, OH, USA) and a selective supplement (SR0183E; Oxoid, Solon, OH, USA). CampyGen (CN0025A; Oxoid, Solon, OH, USA) was introduced to provide microaerophilic conditions desirable for *Campylobacter* isolation. After incubation at 42 °C for 48 h, a loop (10 µL) of each enriched sample was streaked in duplicates onto *Campylobacter* blood-free agar (CM 0739; Oxoid, Solon, OH, USA) with CCDA selective supplement (SR155E; Oxoid, Solon, OH, USA). After incubation at 42 °C for 48 h, plates were examined for morphologically typical of *Campylobacter* colonies (greyish, often with a metallic sheen, flat, and moist with a tendency to spread). Presumptive *Campylobacter* colonies were confirmed by Gram staining, oxidase, and catalase tests. The presumed isolates were further subjected to standard phenotypic tests using the API CAMPY system (bioMérieux, Hazelwood, MO, USA) and *Campylobacter* confirm latex Kit (Scimedx, Denville, NJ, USA).

#### 2.1.3. Detection of Generic *Escherichia coli* and *Escherichia coli* O157:H7

Homogenized samples were incubated for 24 h at 37 °C for pre-enrichment. Approximately, 50 mL of each pre-enriched sample was mixed with 50 mL of double-strength MacConkey broth (Oxoid, Solon, OH, USA) and incubated at 35 °C ± 2 °C for 24 h. After incubation, a loop (10 µL) of each sample was streaked in duplicates onto Eosin-Methylene Blue (EMB) agar plates (BD, Franklin Lakes, NJ, USA) for *Escherichia coli* and Sorbitol MacConkey agar (CM0813; Oxoid, Solon, OH, USA) supplemented with cefixime (50 ng/mL) and potassium tellurite (25 mg/mL) supplement (SR0172E; Oxoid, Solon, OH, USA) for *Escherichia coli O157:H7*. Consequently, inoculated plates were then incubated at 37 °C for 24 h. Isolates with a typical *Escherichia coli* morphology and blue/black with a greenish metallic sheen on EMB were identified as presumptive *Escherichia coli*. *Escherichia coli* colonies were randomly selected and confirmed using the API 20E identification system (bioMerieux, Hazelwood, MO, USA). Colorless, circular, and entire edge colonies with brown centers on the Sorbitol MacConkey agar were identified as presumptive *Escherichia coli* O157:H7. Colonies were further screened for the presence of the O157 antigen by *Escherichia coli* O157 latex test kit (DR0620M, Oxoid, Solon, OH, USA). Presumptive colonies were inoculated onto nutrient agar plates and incubated at 37 °C for 24 h further investigations.

#### 2.1.4. Detection of *Enterococcus* spp.

Each homogenized sample (1 mL) was enriched in 9 mL of sterile Enterococcosel Broth (EB) (BD, Franklin Lakes, NJ, USA) and incubated at 37 °C for 24 h. Next, 10 µL of Enterococcosel broth from positive samples (black) was streaked onto Enterococcosel Agar (EA) plates and incubated at 37 °C for 24 to 48 h. Colonies translucent with brownish-black to black zones on Enterococcosel Agar were identified as *Enterococcus* spp. The phenotypic test for presumptive enterococci isolates was based on morphology and biochemical traits: catalase testing, gram-positive, bile-esculin, and L-pyrrolidonyl-β-naphthylamide activity. In addition, isolates were subjected to the API 20 Strep (bioMérieux, Hazelwood, MO, USA) and results were interpreted using the API web software (version 4.0, bioMe’rieux, Hazelwood, MO, USA).

### 2.2. Bacterial DNA Preparation and Bacteria Confirmation

*Salmonella enterica* serovar, *Escherichia coli* O157:H7, generic *Escherichia coli*, and enterococci isolates were cultivated overnight at 37 °C in tryptic soy broth (TSB; Difco BD). *Campylobacter* isolates were cultivated in Bolton Broth supplemented with 5% Lysed Horse Blood (Oxoid SR0048C) and selective supplement (Oxoid SR0183E). Plates were then incubated at 42 °C for 48 h under previous described micro-aerobic conditions. DNA was extracted from overnight cultures (>5 × 10^6^ cells) using the PureLink Genomic DNA Mini Kit (Life Technologies, Grand Island, NY, USA). DNA concentrations and integrity were determined by using a NanoDrop 2000 (Thermo Scientific, Pittsburgh, PA, USA) and agarose gel electrophoresis, respectively. A PCR CORE Kit (Sigma, St. Louis, MO, USA) was used in this study. Each reaction mixture (25 µL) contained 125 ng of DNA template, 0.5 µM each forward and reverse primers, 400 µM deoxynucleoside triphosphates, 3 mM MgCl2, 2.5 µL of 10x PCR buffer, and 2.5 U Taq DNA polymerase. The sequences of primer pair used for targeting *Salmonella enterica* serovar target gene (*ompC*) was 5′-ATCGCTGACTTATGCAATCG-3 and 5′-CGGGTTGCGTTATAGGTCTG-3′ [16], whereas the primer pair used for targeting *Campylobacter* spp. (16SrRNA) was 5′-ATCT AATGGCTTAACCATTAAAC-3′ and 5′-GGACGGTAACTAGTTTAGTATT-3′ [17]. *Escherichia coli* O157:H7 (*rfbE*) primer pair was 5′- CAGGTGAAGG TGGAATGGTTGTC-3′ and 5′- TTAGAATTGAGACCATCCAATAAG-3′ [18], for *Escherichia coli* (*uidA*) the primer pair was 5′-TGGTAATTACCGACGAAAACGGC-3′ and 5′-TGGTAATT ACCGACGAAAACGGC-3′ [19], and *Enterococcus* spp. (*Tuf*) was 5′-TACTGACAAACCATTCATGATG-3′and 5′AACTTCGTCACCAACGCG AAC-3 [20]. PCR was performed by using a GeneAmp PCR system 2700 thermal cycler (Applied Biosystems, Foster City, CA). Consequently, the PCR products were electrophoresed in agarose gel stained with 0.5 µg/mL of ethidium bromide (Sigma-Aldrich, Madrid, Spain) and photographed under UV light.

### 2.3. Antibiotic Resistant Profiles

Antimicrobial susceptibility was performed in accordance with the standard Kirby disk diffusion method recommended by the Clinical and Laboratory Standards Institute (CLSI, 2016). Antimicrobial disks (BD) used, with strength in parentheses were: amoxicillin/clavulanic acid (AMC; 20/10 µg), cefotaxime (CTX; 30 µg), ceftazidime (CAZ; 30 µg), ceftriaxone (CRO; 30 µg), chloramphenicol (CHL; 30 µg), ciprofloxacin (CIP; 5 µg), erythromycin (ERY; 15 µg), imipenem (IPM; 10 µg), tetracycline (TET; 5 µg), and vancomycin (VAN; 30 µg). A total of 231 bacteria isolates were tested against selected antimicrobials. Briefly, *Salmonella enterica* serovar, *Escherichia coli*, and enterococci overnight cultures were adjusted to 0.5 McFarland standard and spread evenly on Mueller-Hinton (MH) agar plates (Difco, BD). Antibiotic susceptibility disks were then placed on MH plates and incubated for 24 h at 37 °C. For *Campylobacter*, MH broths cultured at 42 °C for 48 h were plated on MH agar supplemented with 5% Lysed Horse Blood. MH plates were then incubated in a microaerophilic atmosphere for 48 h at 42 °C. The zones of inhibition were interpreted according to CLSI guidelines [21]. *Staphylococcus aureus* ATCC 25923 and *Escherichia coli* ATCC 25922 were used as quality control organisms. Reference standard bacterial strains were tested concurrently as controls.

### 2.4. Statistical Analysis

The data was captured in Microsoft Excel^®^ (Microsoft Corporation, Redmond, WA, USA) USA) and analyzed using the analysis of variance of SAS for Windows (version 6.12; SAS Institute, Inc., Cary, NC, USA) and chi-square test. The antibiotic resistance values were expressed as percentages and statistical significance was set at *p*-values of less than 0.05.

## 3. Results and Discussion

### 3.1. Pathogenic Bacteria: *Salmonella enterica* serovar and Campylobacter spp.

*Salmonella enterica* serovar and *Campylobacter* spp. were recovered from edible offal and muscle meats as displayed in Table 1. The prevalence of *Salmonella enterica* serovar at 9.7% was significantly (*p* < 0.05) high as compared to other offal meats, notably none was recovered from beef liver and beef trips. *Salmonella enterica* serovar was also present in chicken wings (4.2%) and ground chicken (6.9%). There was no significant difference of *Salmonella enterica* serovar found in ground beef. However, no significant (*p* > 0.05) differences in the occurrence of this pathogen between edible offal (4.4%) and muscle meats (4.3%).

In a previous study, the prevalence of *Salmonella* in retail chicken and ground turkey was reported at 9.1% and 5.5%, respectively [22]. In addition, Erickson [23] documented a lower (2%) prevalence of *Salmonella* in ground turkey. However, Phan et al. [24] observed a higher *Salmonella* prevalence (21.0%) in chicken meat. According to Wideman et al. [25], the prevalence of Salmonella-contaminated poultry products has dropped significantly due to major and improved alterations in handling of poultry in processing plants. Although none of the beef liver, beef tripe, and ground beef samples were positive for *Salmonella enterica* serovar in our study, this pathogen has been detected at a rate of 3.8% and 2.4% in beef from retail stores and federally inspected establishments, respectively [26]. Murakami et al. [27] also reported 1.5% *Salmonella enterica* serovar occurrence on cattle offal in Fukuoka, Japan.

**Figure 1:**
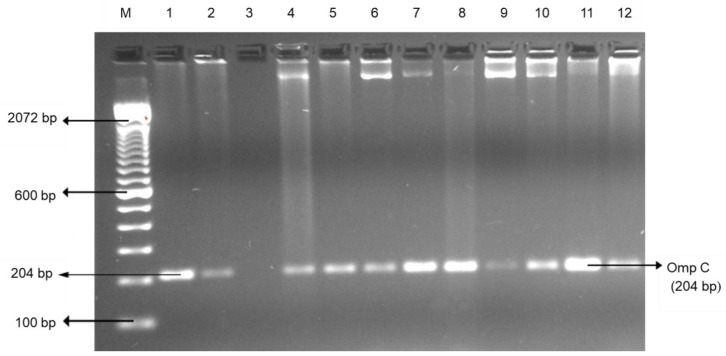
Representative PCR results of *Salmonella enterica* serovar (ompC) isolated from the edible offal and muscle meat. Lane M: 100 bp DNA marker; lane 1–2: positive control; lane 3: negative control; and lane 4–12: positive samples.

In our study, the presence of *Salmonella enterica* serovar in both edible offal and muscle meats was confirmed by PCR (Figure 1).

Generally, as displayed in this study and Zhang et al. [28] investigation, *Salmonella enterica* serovar is frequently found in poultry meats and less in bovine meats. Painter et al. [29] stated that 10–29% of salmonellosis infections in the U.S. are associated with poultry. *Salmonella enterica* serovar is a significant foodborne pathogen in raw meats, mostly in chicken products, and will continue to be a challenge in food chain [30].

**Figure 2:**
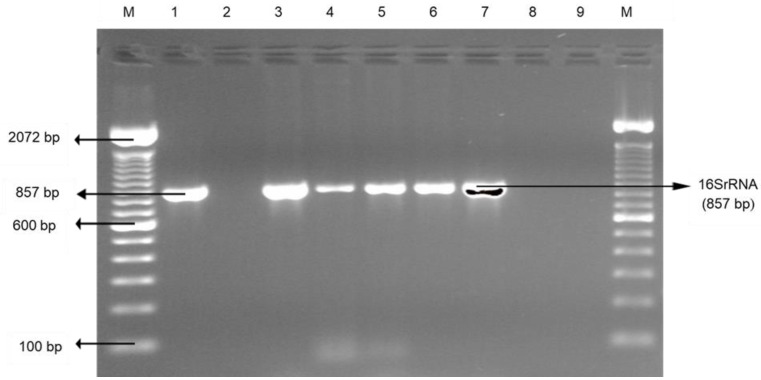
Representative PCR results of *Campylobacter* (16SrRNA) isolated from the edible offal and muscle meat. Lane M: 100 bp DNA marker; lane 1: positive control; lane 2: negative control; and lane 3–9: samples (lane 3–7: positive samples; lane 8–9: negative sample).

Low *Campylobacter* frequency was observed in ground beef (2.3%) and the ground chicken (1.4%) as shown in Table 1. Overall, no significant difference was observed in *Campylobacter* occurrence between edible offal and muscle meats. However, specifically beef liver (6.9%) showed a significantly higher prevalence (*p* < 0.05) than ground chicken (1.4%) and ground beef (2.3%). Trokhymchuk et al. [31] reported a higher *Campylobacter* rate (16.2%) in retail ground beef than in our study (2.3%). Our results also show 6.9% of *Campylobacter* on the beef liver, relatively in agreement with Enokimoto et al. [32] study that displayed a 5% prevalence. However, other studies have reported higher *Campylobacter* occurrences of 78% [33] and 69% [34]. The elevated *Campylobacter* incidence in retail beef liver might be due to cross-contamination since they are recovered from several cows and amassed together [33]. Ghafir et al. [35] also suggested that the raised level of *Campylobacter* recovery from liver may be due to elevated moisture content, which provides protection to the foodborne pathogen. The risk of *Campylobacter* in beef liver could also be magnified by preparing lightly to evade charring and undesired taste [35]. According to Vipham et al. [36], retail ground beef storage environment could also favor *Campylobacter* survival. In the current study, *Campylobacter* spp. on edible offal and muscle meats was confirmed as displayed in Figure 2.

**Figure 3:**
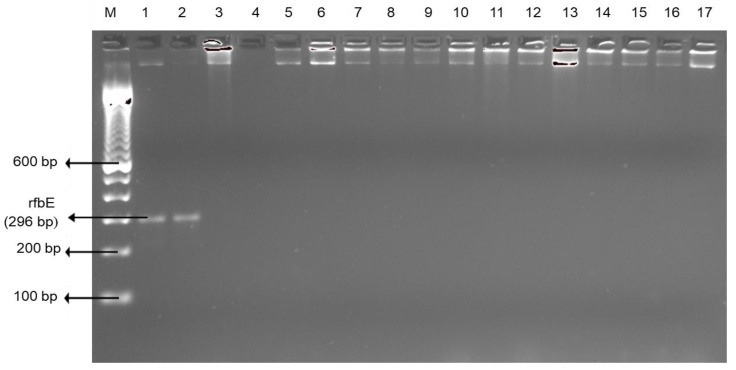
Representative PCR results of E. coli O157:H7 (rfbE) isolated from the edible offal and muscle meat. Lane M: 100 bp DNA marker; lane 1–2: positive control; lane 3: negative control; and lane 4–17: samples. All samples were negative for *E. coli* O157:H7.

Although there have been improvements in handling practices, retail meat and liver products may be tainted with *Campylobacter* at the slaughterhouse [37]. *Campylobacter* has the potential to survive severe environments through utilizing multiple survival mechanisms [38]; hence, potential for future outbreaks owing to the inclination for undercooked liver [39] and other meat products. It is also documented that Campylobacteriosis outbreaks have been linked to contaminated retail liver products worldwide [40,41]. In our study, there were no *Escherichia coli* O157:H7 positive edible offal or muscle meats (Figure 3).

### 3.2. Indicator Bacteria: Escherichia coli and enterococci

*Escherichia coli* and enterococci were observed in all edible offal and muscle meats as displayed in Table 1. Overall, muscle meats presented significantly (*p* < 0.05) higher *Escherichia coli* occurrence (89.4%) than in edible offal (79.4%) (Table 1). Our findings are supported by the Food Authority reporting a higher occurrence of *Escherichia coli* in beef cuts than beef offal [42]. In reference to edible offal, the frequency of *Escherichia coli* was significantly (*p* < 0.05) higher in chicken liver (93.1%) as compared to beef tripe (72.7%), and beef liver (63.6%). However, in another report, *Escherichia coli* was isolated at 40% from beef liver [43]. From the statistical analysis, it can be concluded that prevalence levels of *Escherichia coli* were not significantly (*p* > 0.05) different in the ground chicken (94.4%) and chicken wings (93.1%).

**Figure 4:**
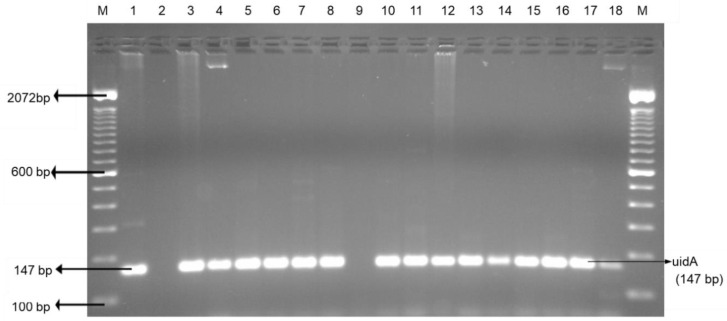
Representative PCR results of *E. coli* (uidA) isolated from the edible offal and muscle meat. Lane M: 100 bp DNA marker; lane 1: positive control; lane 2: negative control; and lane 3–18: samples; (lane 3–8 and lane 10–18: positive samples; lane 9: negative sample).

**Figure 5:**
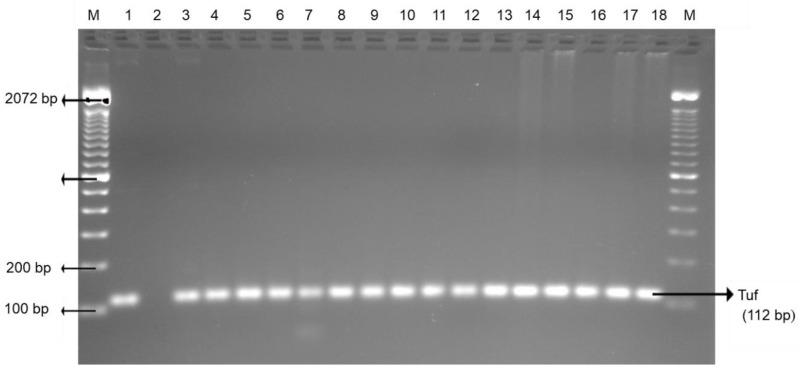
Representative PCR results of enterococci (Tuf) isolated from the edible offal and muscle meat. Lane M: 100 bp DNA marker; lane 1: positive control; lane 2: negative control; and lane 3–18: positive samples.

Chicken wings and ground chicken presented the highest enterococci (100%), followed by chicken liver (97.2%), beef liver, ground beef (81.8%), and beef tripe (79.5%). Ground beef, beef tripe, and beef liver showed significantly (*p* < 0.05) lower enterococci prevalence than in chicken liver, chicken wing, and ground chicken (Table 1). The occurrence of enterococci in retailed offal and muscle meats is a health concern as it is the third primary source of nosocomial infections in the United States [44]. Improper handling practices of enterococci contaminated meat may result to nosocomial infection. *Escherichia coli* and enterococci incidence in foods is reflected as an indicator of fecal contamination and occurrence of pathogenic bacteria in the interrelated food items [45]. Figure 4 and Figure 5 are PCR confirmations of *Escherichia coli* and enterococci occurrence in both edible offal and muscle meats, respectively.

### 3.3. Antimicrobial Drug Resistance in Pathogenic Bacteria

Detailed presentations of antimicrobial-resistant bacteria in edible offal and muscle meats are shown in Table 2. Phenotypic screening of antimicrobial resistance among pathogenic bacteria in our study revealed resistance to essential antimicrobials in human medicine [46]. *Salmonella enterica* serovar recovered from edible offal and muscle meats demonstrated resistance to at least six and eight antibiotics, respectively. *Salmonella enterica* serovar recovered from muscle and offal meats was multidrug-resistant (MDR). *Salmonella enterica* serovar significantly (*p* < 0.05) showed high resistance to vancomycin (100% and 100%), erythromycin (100% and 100%), and tetracycline (85.7% and 100%) as compared to chloramphenicol (71.4% and 62.5%), ceftriaxone (57.1% and 62.5%), and cefotaxime (42.9% and 62.5%) in edible offal and muscle meats, respectively.

Our findings are in agreement with previous a study [47] that documented *Salmonella* resistance to erythromycin, tetracycline, and amoxicillin/clavulanic acid in retail meats. Resistance to ceftriaxone and cefotaxime was also exhibited by *Salmonella enterica* serovar isolates (*n* = 7) in our study. Hence, a concern since these drugs are first line therapy for salmonellosis and an option to treat antimicrobial resistant *Salmonella* in human medicine. Resistance to ciprofloxacin and imipenem was not observed among *Salmonella* isolates. In a previous report, 92 people in 29 states in the U.S. were infected with MDR *Salmonella enterica* serovar traced to raw chicken products [48]. The occurrence of *Salmonella enterica* serovar resistance to clinically important drugs is a challenge; it could translate to the constricted option of Salmonellosis treatment. Salmonella gastroenteritis is typically a self-limiting disease; however, antibiotics are suggested for patients at a greater threat for invasive diseases [49].

No significant (*p* > 0.05) difference in antimicrobial resistance was observed in *Campylobacter* (Table 2) isolates (*n* = 3) in all antibiotics tested in this study. *Campylobacter* showed high resistance to cefotaxime (100%), ceftriaxone (100%), erythromycin (100%), and vancomycin (100%). Our results are in agreement with Ge et al. [50] study that noted resistance to tetracycline (82%), erythromycin (54%), and ciprofloxacin (35%) among *Campylobacter* isolates from retail chicken. *Campylobacter* resistance for other antibiotics was noted to tetracycline (33.3% and 0%) and ciprofloxacin (100% and 50%) for offal and muscle meats, respectively. Research has revealed a major link between *Campylobacter* strains presenting resistance to tetracycline and ciprofloxacin [51].

Resistance to erythromycin and ciprofloxacin is a major health concern since these drugs are used in the treatment of *Campylobacter* infections [52]. The upsurge prevalence of ciprofloxacin-resistant *Campylobacter* in retail poultry meat was also reported in Denmark [53] Spain, Germany, Italy, Holland, and Austria [54]. Nisar et al. [55] also documented *Campylobacter* resistance to ciprofloxacin. None of the *Campylobacter* isolates in this study were resistant to amoxicillin/clavulanic acid, ceftazidime, and imipenem. To avert antibiotic-resistant *Campylobacter* in edible offal and other meats from infecting consumers, chefs and other food preparers must adhere to basic food hygiene practices while handling and preparing meats. Suzuki and Yamamoto [56] reported that *Campylobacter* is frequently associated with the handling and consumption of meat products, especially raw poultry.

### 3.4. Antimicrobial Drug Resistance in Escherichia coli and Enterococci

Antimicrobial resistance of *Escherichia coli* from edible offal and muscle meats was observed to clinically important antimicrobials, notably amoxicillin/clavulanic (100% and 92.2%), ceftriaxone (73.6% and 54.9%), and ciprofloxacin (34% and 27.5%), respectively. *Escherichia coli* resistance to amoxicillin/clavulanic acid was significantly (*p* < 0.05) higher as compared to ceftriaxone resistance in edible offal and muscle meats (Table 2). *Escherichia coli* resistance to fluoroquinolones is disturbing, especially ciprofloxacin is important for treating *Escherichia coli* infections in both humans and animals [57]. *Escherichia coli* from edible offal and muscle meats significantly (*p* < 0.05) presented high levels of resistance to erythromycin (100% and 100%), tetracycline (100% and 100%), and vancomycin (100% and 100%) as compared to ciprofloxacin (34% and 27.5%), and ceftazidime (17% and 17.7%), respectively. Notably, only one *Escherichia coli* isolate from muscle meat was resistant to imipenem.

Our findings suggest that antibiotic-resistant *Escherichia coli* was prevalent in retail beef liver. This is a health risk since edible offal is often undercooked to preserve nutrient content [58]. Epidemiological records have indicated food poisoning linked to *Escherichia coli* O157:H7 from beef liver in Japan [59]. Our findings show that edible offal is contaminated with antimicrobial-resistant bacteria, therefore it is essential for consumers to adhere to food safety practices while preparing offal dishes. The presence of multidrug-resistant *Escherichia coli* on meats is an impending challenge in infectious diseases. *Escherichia coli* capability to survive and attain resistance in the guts of animals has been labeled as a sentinel organism in antimicrobial resistance surveillance programs [60].

Overall, enterococci from edible offal and muscle meats significantly (*p* < 0.05) exhibited high resistance to ceftriaxone (98.1% and 94.4%), cefotaxime (96.2% and 90.6%), and ceftazidime (96.2% and 90.7%) as compared to amoxicillin/clavulanic acid (0% and 1.9%), respectively (Table 2). enterococci from edible offal and muscle meats also demonstrated resistance to ciprofloxacin (60.4% and 90.7%) and vancomycin (75.5% and 83.3%), respectively. Ciprofloxacin and vancomycin are antimicrobials of great importance in human medicine [61]. According to Klare et al. [62] and from a medical angle, vancomycin is an essential therapeutic drug against multidrug-resistant enterococci. Despite the fact enterococci are reflected as opportunist pathogens, our findings reflect them as potential reservoirs of antimicrobial resistance. Therefore, it is a health risk for the occurrence of multidrug-resistant enterococci in retail offal and muscle meats. Jackson et al. [63] report that enterococci is a reservoir of antimicrobial resistance genes and has the potential to transfer these genes to other bacteria in the environment. According to Carvalho et al. [64], *Escherichia coli* and enterococci are reservoirs of antimicrobial-resistant genes that could be disseminated to pathogenic bacteria in the environment. It is essential to place focused effective control measures at decreasing enterococcus in retail meats, since it is a leading cause of nosocomial infections [65]. Agglutination test displayed higher prevalence of both pathogenic and indicator bacteria than PCR method.

### 3.5. Multidrug Resistant Patterns of Pathogenic and Indicator Bacteria

In our study, both pathogenic and indicator bacteria were MDR. According to Nguyen et al. [66], MDR isolate displays resistance to three or more classes of antibiotics. A total of 41 multidrug-resistant profiles were presented among pathogenic and indicator bacteria from retail offal and muscle meats (Table 3). *Salmonella* from offal and muscle meats displayed AMC-CTX-CRO-CHL-ERY-TET-VAN (0; 1), CTX-CAZ-CRO-CHL-ERY-TET-VAN (0; 1) and CTX-CRO-CHL-ERY-TET-VAN (2; 2) resistance patterns, respectively (number of isolates in parenthesis). CTX-CRO-CHL-ERY-TET-VAN and ERY-TET-VAN were the most prevalent (*p* < 0.05) antimicrobial resistance patterns in *Salmonella* (Table 3). According to our results, it is clear that both edible and muscle meats are potential sources of MDR *Salmonella*. MDR *Salmonella* is an emerging public health challenge worldwide and has repetitively been reported in food animals, a consequential exposure to humans through the food chain [66].

Dissimilar drug resistance patterns including CTX-CRO-CHL-ERY-VAN (0; 1), CRO-CHL-ERY-VAN (0; 1) were also recorded for *Campylobacter* from offal and muscle meats, respectively. Our results displayed MDR *Campylobacter* isolates, a concern and challenge in campylobacteriosis management [67]. None of the antimicrobial resistance patterns were predominant (*p* > 0.05) over other patterns among *Campylobacter* isolates (Table 3).

Overall, *Escherichia coli* displayed 10 multidrug resistance patterns. AMC-CHL-ERY-TET-VAN (23; 15), AMC-ERY-TET-VAN (12; 25) were the common phenotypes displayed by *Escherichia coli* on offal and muscle meats, respectively. Other *Escherichia coli* patterns included AMC-CAZ-CHL-ERY-TET-VAN (9; 2) and AMC-CRO-CHL-ERY-TET-VAN (1; 1) in offal and muscle meats, respectively. AMC-CHL-ERY-TET-VAN was the most significant (*p* < 0.05) antimicrobial resistance pattern among *Escherichia coli* isolates from edible offal. On the other hand, *Escherichia coli* from muscle meat displayed AMC-ERY-TET-VAN pattern which was significantly dominant (*p* < 0.05) compared to other patterns (Table 3). The rise of MDR *E. coli* is disturbing, especially strains causing urinary tract infections [68].

Our findings reveal that Enterococci exhibited the most (*n* = 22) multidrug-resistant patterns. CTX- CAZ-CRO-CHL-CIP-TET (0; 2), CTX-CAZ- CHL- CIP- ERY- TET (0; 2), CTX-CAZ-CRO-TET (15; 6), CTX-CAZ-CRO-ERY-TET (10; 9), and CTX-CAZ-CRO-ERY-TET-VAN (6; 8) patterns were displayed for enterococci from offal and muscle meats, respectively. Among enterococci isolates, CTX-CAZ-CRO-TET was observed as the most significant (*p* < 0.05) antimicrobial resistance pattern in edible offal and CTX-CAZ-CRO-ERY-TET-VAN for muscle meats. Enterococci infections are a concern to public health owing to the struggle in controlling them with antimicrobials. According to Weiner et al., [69], vancomycin-resistant enterococci (VRE) are utmost baffling to treat, as some exhibit resistance to all accessible antimicrobials used in medical practice. Chloramphenicol resistance was common as indicated by resistance patterns (Table 3) and according to Whichard et al. [70], chloramphenicol is a unique drug that can be applied to monitor and predict multidrug resistance of foodborne pathogens from animals and retail meats. To prevent foodborne illnesses, consumers must embrace hygienic practices, which include recommended storage and cooking temperatures for all meat types [71]. The findings of our study may assist in tracking the emergence of new resistance patterns among pathogenic and indicter organisms in edible offal and also in other meats.

## 4. Conclusions

This study demonstrated that certain isolates of *Salmonella enterica* serovar, *Campylobacter* spp., *Escherichia coli*, and enterococci from edible offal as well as muscle meats were resistant to “critically important antimicrobials (AMC, CTX, CAZ, CRO, CIP, ERY, IPM, and VAN)” or “highly important antimicrobials (CHL and TET)” used in human medicine [72]. The presence of multidrug-resistant pathogenic and indicator bacteria in edible offal suggests periodic monitoring of these meats. Our data reinforced the need to better recognize the role of retail edible offal and muscle meats as sources of MDR bacteria. This data is desirable for improving the food safety of variety meats which are important in southern cuisine and increasing in popularity in several cities as a result of immigrants’ influence.

## Figures and Tables

**Table 1:** Prevalence (%) of pathogenic and indicator bacteria in retail edible offal and muscle meats.

Bacterial Species ^A^	No. (%) of Edible Offal ^B^				No. (%) of Muscle Meat ^B^			
	CL (*n* = 72)	BL (*n* = 44)	BT (*n* = 44)	Total (*n* = 160)	CW (*n* = 72)	GC (*n* = 72)	GB (*n* = 44)	Total (*n* = 188)
*Salmonella enterica* serovar	7 (9.7) ^ay^	0 (0.0) ^cy^	0 (0.0) ^cy^	7 (4.4) ^aby^	3 (4.2) ^aby^	5 (4.2) ^aby^	0 (0.0) ^cy^	8 (4.3) ^aby^
*Campylobacter*	0 (0.0) ^by^	3 (6.9) ^ay^	0 (0.0) ^by^	3 (1.9) ^aby^	0 (0.0) ^by^	1 (1.4) ^aby^	1 (2.3) ^aby^	2 (1.1) ^aby^
*Escherichia coli*	67 (93.1) ^ax^	28 (63.6) ^bx^	32 (72.7) ^abx^	127 (79.4) ^abx^	67 (93.1) ^ax^	68 (94.4) ^ax^	33 (75.0) ^abx^	168 (89.4) ^ax^
enterococci	70 (97.2) ^ax^	36 (81.8) ^bx^	35 (79.5) ^bx^	141 (88.1) ^bx^	72 (100.0) ^ax^	72 (100.0) ^ax^	36 (81.8) ^bx^	180 (95.7) ^ax^

^A^ Bacterial species isolated from the edible offal and muscle meat. ^B^ Edible offal included chicken liver (CL), beef liver (BL), and beef tripe (BT); Muscle Meat included chicken wing (CW), ground chicken (GC), and ground beef (GB). ^a–c^ Mean percentages in the same row followed by different letters are significantly different (*p* < 0.05). ^x–y^ Mean percentages in the same column followed by different letters are significantly different (*p* < 0.05).

**Table 2:** Prevalence of antibiotic resistant bacteria from the edible offal and muscle meats.

Bacterial Species ^A^	Isolates (n = 116)	No. (%) of Bacteria Isolates in the Edible Offal Resistant to Antimicrobial Agents ^B^									
		AMC	CTX	CAZ	CRO	CHL	CIP	ERY	IPM	TET	VAN
**Offal meats**											
*Salmonella enterica* serovar	7	0 (0.0) ^c^	3 (42.9) ^ab^	0 (0.0) ^c^	4 (57.1) ^ab^	5 (71.4) ^a^	0 (0.0) ^c^	7 (100) ^a^	0 (0.0)^c^	6 (85.7) ^a^	7 (100) ^a^
*Campylobacter*	3	0 (0) ^a^	3 (100) ^a^	0 (0) ^a^	3 (100) ^a^	0 (0) ^a^	3 (100) ^a^	3 (100) ^a^	0 (0) ^a^	1 (33.3) ^a^	3 (100) ^a^
*Escherichia coli*	53	53 (100) ^a^	39 (73.6) ^ab^	9 (17) ^c^	39 (73.6) ^ab^	36 (67.9) ^ab^	18 (34) ^bc^	53 (100) ^a^	0 (0) ^c^	53 (100) ^a^	53 (100) ^a^
*Enterococci*	53	0 (0) ^c^	51 (96.2) ^a^	51 (96) ^a^	52 (98) ^a^	9 (17) ^bc^	32 (60) ^ab^	51 (96.2) ^a^	0 (0) ^c^	49 (92.5) ^a^	40 (75.5) ^ab^
Total	116	53 (46) ^bc^	96 (83) ^ab^	60 (52) ^bc^	98 (84.5) ^ab^	50 (43.1) ^bc^	53 (46) ^bc^	114 (98.3) ^a^	0 (0) ^c^	109 (94) ^a^	103 (88.8) ^ab^
**Muscle meats**											
*Salmonella enterica* serovar	8	2 (25) ^bc^	5 (62.5) ^ab^	2 (25) ^bc^	5 (62.5) ^ab^	5 (62.5) ^ab^	0 (0.0) ^c^	8 (100) ^a^	0 (0) ^c^	8 (100) ^a^	8 (100) ^a^
*Campylobacter*	2	0 (0)^a^	2 (100) ^a^	0 (0) ^a^	2 (100) ^a^	2 (100) ^a^	1 (50) ^a^	2 (100) ^a^	0 (0) ^a^	0 (0) ^a^	2 (100) ^a^
*Escherichia coli*	51	47 (92.2) ^a^	26 (51) ^ab^	9 (17.7) ^c^	28 (55) ^ab^	28 (55) ^ab^	14 (26) ^bc^	51 (100) ^a^	1 (2) ^c^	51 (100) ^a^	51 (100) ^a^
*Enterococci*	54	1 (1.9) ^c^	50 (92.6) ^a^	49 (90.7) ^a^	51 (94.4) ^a^	27 (50) ^ab^	49 (90.7) ^a^	53 (98.1) ^a^	0 (0) ^c^	54 (100) ^a^	45 (83.3) ^a^
Total	115	50 (43.5) ^bc^	83 (71.2) ^b^	60 (52.2) ^bc^	86 (74.8) ^b^	62 (53.9) ^bc^	64 (55.7) ^bc^	114 (99.1) ^a^	1 (0.9) ^c^	113 (98.3) ^a^	106 (92.2) ^a^

^A^ Bacterial species isolated from the edible offal. ^B^ Ten selected antibiotics for antimicrobial susceptibility test: Amoxicillin/Clavalanic acid (AMC); Cefotaxime (CTX); Ceftazidime (CAZ); Ceftriaxone (CRO); Chloramphenicol (CHL); Ciprofloxacin (CIP); Erythromycin (ERY); Imipenem (IPM); Tetracycline (TET); and Vancomycin (VAN). ^a–c^ Mean percentages in the same row followed by different letters are significantly different (*p* < 0.05).

**Table 3:** Multidrug resistance patterns of pathogenic and indicator bacteria in edible offal and muscle meat.

Bacterial Species ^A^	Antibiotic Resistance Profiles ^B^	No. (%) of Edible Offal Isolates ^C^ (n = 116)	No. (%) of Muscle Meat Isolates ^C^ (n = 115)
*Salmonella enterica* serovar (n = 15)	AMC, CTX, CRO, CHL, ERY, TET, VAN	0 (0.0) ^c^	1 (0.9) ^c^
	AMC, CHL, ERY, TET, VAN	0 (0.0) ^c^	1 (0.9) ^c^
	CTX, CAZ, CRO, CHL, ERY, TET, VAN	0 (0.0) ^c^	1 (0.9) ^c^
	CTX, CAZ, ERY, TET, VAN	0 (0.0) ^c^	1 (0.9) ^c^
	CTX, CRO, CHL, ERY, TET, VAN	2 (1.7) ^bc^	2 (1.7) ^bc^
	CTX, CHL, ERY, TET, VAN	1 (0.9) ^c^	0 (0.0) ^c^
	CRO, CHL, ERY, TET, VAN	1 (0.9) ^c^	0 (0.0) ^c^
	ERY, TET, VAN	2 (1.7) ^bc^	2 (1.7) ^bc^
	ERY, VAN	1 (0.9) ^c^	0 (0.0) ^c^
*Campylobacter* (n = 5)	CTX, CRO, CHL, ERY, VAN	0 (0.0) ^c^	1 (0.9) ^c^
	CRO, CHL, ERY, VAN	0 (0.0) ^c^	1 (0.9) ^c^
	CRO, ERY, VAN	1 (0.9) ^c^	0 (0.0) ^c^
	CIP, ERY, VAN	1 (0.9) ^c^	0 (0.0) ^c^
	ERY, VAN	1 (0.9) ^c^	0 (0.0) ^c^
*E. coli* (n = 104)	AMC, CTX, CRO, CHL, ERY, TET, VAN	1 (0.9) ^c^	0 (0.0) ^c^
	AMC, CTX, ERY, TET, VAN	0 (0.0) ^c^	1 (0.9) ^c^
	AMC, CAZ, CRO, CHL, ERY, TET, VAN	2 (1,7) ^bc^	2 (1.7) ^bc^
	AMC, CAZ, CHL, ERY, TET, VAN	9 (7.8) ^bx^	2 (1.7) ^bcy^
	AMC, CRO, CHL, ERY, TET, VAN	5 (4.3) ^bc^	1 (0.9) ^c^
	AMC, CHL, CIP, ERY, TET, VAN	1 (0.9) ^c^	0 (0.0) ^c^
	AMC, CHL, ERY, TET, VAN	23 (19.8) ^ax^	15 (13.0) ^aby^
	AMC, ERY, IPM, TET, VAN	0 (0.0) ^c^	1 (0.9) ^c^
	AMC, ERY, TET, VAN	12 (10.3) ^aby^	25 (21.7) ^ax^
	ERY, TET, VAN	0 (0.0) ^c^	4 (3.5) ^bc^
*Enterococci* (n = 107)	AMC, CTX, CRO, ERY, TET, VAN	0 (0.0) ^c^	1 (0.9) ^c^
	CTX, CAZ, CRO, CIP, ERY, TET, VAN	1 (0.9) ^c^	5 (4.3) ^bc^
	CTX, CAZ, CRO, CHL, CIP, TET	0 (0.0) ^c^	2 (1.7) ^bc^
	CTX, CAZ, CRO, CIP, ERY, TET	2 (1.7) ^bc^	0 (0.0) ^c^
	CTX, CAZ, CRO, CIP, TET, VAN	0 (0.0) ^bc^	2 (1.7) ^bc^
	CTX, CAZ, CRO, ERY, TET, VAN	6 (5.2) ^b^	8 (7.0) ^b^
	CTX, CAZ, CHL, CIP, ERY, TET	0 (0.0) ^c^	2 (1.7) ^bc^
	CTX, CAZ, CRO, CIP, TET	1 (0.9) ^c^	0 (0.0) ^c^
	CTX, CAZ, CRO, ERY, TET	10 (8.6) ^bx^	9 (7.8) ^by^
	CTX, CAZ, CRO, TET, VAN	2 (1.7) ^bc^	7 (6.1) ^b^
	CTX, CAZ, CRO, ERY	2 (1.7) ^bc^	0 (0.0) ^c^
	CTX, CAZ, CRO, TET	15 (12.9) ^abx^	6 (5.2) ^by^
	CTX, CAZ, CRO, VAN	3 (2.6) ^bc^	0 (0.0) ^c^
	CTX, CAZ, ERY, TET	2 (1.7) ^bc^	0 (0.0) ^c^
	CTX, CAZ, CRO	0 (0.0) ^c^	3 (2.6) ^bc^
	CTX, CAZ, CIP	2 (1.7) ^bc^	0 (0.0) ^c^
	CAZ, CRO, ERY, TET, VAN	0 (0.0) ^c^	1 (0.9) ^c^
	CAZ, CRO, ERY, TET	2 (1.7) ^bc^	0 (0.0) ^c^
	CAZ, ERY, TET, VAN	1 (0.9) ^c^	0 (0.0) ^c^
	CAZ, CRO, TET	0 (0.0) ^c^	1 (0.9) ^c^
	CAZ, CRO	0 (0.0) ^c^	3 (2.6) ^bc^
	CAZ, TET	2 (1.7) ^bc^	0 (0.0) ^c^
	CHL, TET, VAN	1 (0.9) ^c^	0 (0.0) ^c^
	ERY, TET, VAN	1 (0.9) ^c^	2 (1.7) ^bc^
	ERY, VAN	0 (0.0) ^c^	2 (1.7) ^bc^

^A^ Bacterial species isolated from the edible offal and muscle meat. ^B^ Ten selected antibiotics for antimicrobial susceptibility test: Amoxicillin/Clavulanic acid (AMC); Cefotaxime (CTX); Ceftazidime (CAZ); Ceftriaxone (CRO); Chloramphenicol (CHL); Ciprofloxacin (CIP); Erythromycin (ERY); Imipenem (IPM); Tetracycline (TET); and Vancomycin (VAN). ^C^ Edible offal meats included chicken liver (CL), beef liver (BL), and beef tripe (BT); Muscle Meats included chicken wing (CW), ground chicken (GC), and ground beef (GB). ^a–c^ Mean percentages in the same column followed by different letters are significantly different (*p* < 0.05). ^x–y^ Mean percentages in the same row followed by different letters are significantly different (*p* < 0.05).
